# Application of nanoparticles in Dentistry

**DOI:** 10.6026/97320630019014

**Published:** 2023-01-31

**Authors:** Sheloni Missier Mary, Mahesh Ramakrishnan, Saravana Dinesh Sudalaimani Paulpandian, Shanmugam Rajeshkumar, Jebilla Pringle

**Affiliations:** 1 Saveetha Dental College and Hospital, SIMATS, Saveetha University, Chennai, Tamilnadu, India; 2 Department of Orthodontics, Rajas Dental College and Hospital, Kavalkinaru, Tamilnadu, India

**Keywords:** Nanoparticles, Orthodontics, Antimicrobial property

## Abstract

Nanoparticles(NPs) are of particle sizes lesser than 100nm and are insoluble the field which deal with the handling of these particles is coined as "Nanotechnology" as their particle size is smaller, they can penetrate easily therefore they are applied in various medical fields, drug delivery and in dentistry as they have antimicrobial property, reduces friction, anti-inflammatory and antioxidant property. Many studies have been done to evaluate its application and its cytotoxicity by varying its concentration and various studies have been done to evaluate its physical property. Therefore, it is of interest to describe concepts of nanoparticles, mode of action, tissue reaction and its application in orthodontics.

## Background:

Nanotechnology is considered to be the 21st century current technology based on its economic and scientific potential which concerns the structures on a Nano scale. According to the British Standards Institution nanoparticles are defined by its particle size or diameter which is measured in nanoscale [1]. Whereas, nanotechnology is the art of science in which the size of the particle is measured in about one billionths of a nanometer, which is roughly the size of two to three atoms [2]. Nanodentistry is the recent development of science and technology by incorporating nanomaterials, tissue engineering and nanorobotics in dental materials to maintain good oral health due to its antimicrobial property [3]. Engineered nanoparticles are nanoparticles which are produced intentionally with the same characteristics, particle size of 1 to 100nm and similar properties which are different from non-nanoscale particles but with the same chemical component [4]. There are various advancements in material sciences and technology producing beneficial results, one of such kind is nanotechnology, which basically startedby surface coatings later lead to the formation of newer material.

## Various Types of Nanoparticles:

Nanoparticles are basically categorized according to their morphology, texture, composition, uniformity, dimension and agglomeration. They are of various types such as Nano pores, Quantum dots, Nanotubes, Nano shells, Nanorods, Dendrimers, Fullerenes, Liposomes, Nano spheres, Nano capsules, Nano rings, Nano belts and Nano wires [5]. Some of the effectively utilized nanoparticles in various fields are as follows.

## Gold:

With the use of Nano stencilled RGD gold nanoparticles detection of DNA has been made easy by identifying the proteins thereby using it in various cell culture and tissue engineering studies, it also plays a major role in diagnosis of cancer [6-7].

## Titanium Dioxide:

In biomaterials in-order to enhance antimicrobial property titanium-dioxide nanoparticles compound is added in addition to it due to its texture, colour, reduced toxicity, increased stability, its catalytic effect and reduced cost has been an added advantage which has made it an appropriate additive to be used in dental materials [8].

## Silver:

Silver nanoparticles are known for its antimicrobial property as they are seen effective against spores, bacteria and viruses therefore they are employed in various fields like water treatment, in sunscreen lotions, textile industries [9,10,10-12], in dentistry it is used for the fabrication of newer materials such as cements, resins etc due to its antimicrobial property. Due to its implications in various fields in order to reduce its cytotoxicity various plants have been used for green synthesis of the silver nanoparticle using plant extracts such as *Capsicum annuum* [13,14] and *Azadirachta indica* [10].

## Copper:

Copper nanoparticles have various properties such as antifungal, antibacterial, optical, electrical and catalytic properties therefore it can be implemented in various medical fields by infusing them using micro emulsion technique.

## Alloys:

The alloy nanoparticles have different structural properties than bulk samples [15]. Bimetallic alloy nanoparticles show better beneficial properties compared to the other metallic NPs as the properties are influenced by both the metals [16]. In certain studies it showed that silver flakes are widely used due to high electrical conductivity property compared to other metal fillers whereas oxides have improved conductivity compared to silver [17].

## Zinc:

Zinc nanoparticles inhibit the metabolism of sugar by displacing the magnesium ions and disrupting the enzymes thereby inhibiting the formation of biofilms [18]. It is mostly of biomedical usage as they are biocompatible and poses the additional properties like antifungal, ultra-violet filtering properties, anti-corrosive and antibacterial property.

## Magnetic nanoparticles:

Nanoparticles have been widely used in various fields due to its additional properties with its invention drug delivery, treatment of cancer has been made easy, one among them is Fe2O3 (maghemite) and Fe3O4 (magnetite) both are magnetic nanoparticles they are used mostly in MRI (magnetic resonance imaging), gene therapy, stem cell manipulation, DNA profiling and sorting of stem cells [19].

## Quaternary Ammonium Nanoparticles:

Quaternary nanoparticles are known for its antimicrobial property in addition to it they have hydrophobic nature and cationic surface charge property thereby enhancing its action. Due to its antimicrobial property in recent fields they are being incorporated into composite resins.

## Chitosan:

Chitin is a polymer which is naturally available in the aquatic exoskeletons of shelled creatures as they are positively charged they are ideal in incorporating in drug delivery.

## Dental Applications of Nanoparticles:

As the particle size is smaller nanoparticles have better penetration and causes effective cell-lysis therefore it is been incorporated in various fields of dentistry such as treatment of cancer, cancer diagnosis, local anaesthesia and hypersensitivity. Nanoparticles are available in various forms among which nano-fibers and nano-needles are been used in wound dressings [20]. There are various properties such as antibacterial, anti-adhesive, cationic and biocidal property due to the advantage of smaller particle size and easy penetration they can spread over a larger surface area compared to other particles and can interact with the microbial membranes thereby inhibiting the formation of biofilm which indirectly help in the reduced caries formation. With the invention of dentifrobots new treatment options have been introduced which provides permanent solution to treat hypersensitivity, renaturalization of teeth, immediate results post orthodontic treatment and enamel remineralization thereby aid in proper oral hygiene maintenance. As the particle size of the nanoparticles is smaller they have various applicants by incorporating in the dental materials, coating of devices, topical agents [21, 22]. Due to its antimicrobial property silver nanoparticles are been incorporated in composites whereas silver zeolite are being incorporated in toothpaste and mouth washes [23]. Among various other nanoparticles zinc and silver particles have better antimicrobial activity compared to others as their particles are smaller in size compared to other nanoparticles therefore it penetrates the cell membrane causing cell lysis [24]. Dentures, retainers, removable appliances are more prone to bacterial colonisation which leads to oral disease due to infections in-order to reduce the bacterial colonisation lesser percentage of silver and zinc nanoparticles are being incorporated in the polymer of the acrylic resin and acrylised and used in removable appliances and acrylic base plates for dentures [25]. In restorative dentistry in order to improve the quality of treatment by remineralization of the tooth due to the antimicrobial property of the nanoparticles they are being incorporated in cements, sealers, fiber post, pit and fissure sealants and cavity liners [26]. Mixing of alginate impression powders with water containing silver hydrosol can be considered to create an impression material with an antimicrobial property, reducing microbial cross contamination to the poured stone model from the infected impression [27]. Due to the additional property of nanoparticles they are been added to impression material as they hydrophilic in nature and enhance the flow of the impression material nano-fillers are being incorporated in the poly viny siloxane materials as the give better detailing of the teeth [28].

## Orthodontic implications of Nanoparticles:

## Orthodontic Brackets:

In 2012 UC3M found a nanoparticle containing alumina and polysulphone which was biocompatible, increased strength and reduced friction therefore due to these properties they were used for fabricating orthodontic brackets as they didn't increase the thickness of the bracket and maintain its transparency.

## Doping of Nitrogen in Titanium dioxide brackets:

Nitrogen doped titanium dioxide when used to coat brackets exert antimicrobial property although they form hydroxide free radicals (OH), superoxide ions (O2), peroxyl radicals (HO2) which in turn reacts with the protein, enzymes, necrosed tissue, and lipids causing a sequence of oxidative reactions due to which these brackets have reduced bond strength, these nanoparticle was incorporated in the composite paste (Transbond XT) and it was found that they had the same amount of antibacterial effect in whatever concentration they were used [29].

## Friction reducing nano-coated arch wires and brackets:

There are some factors which impede the treatment during the retraction, alignment of orthodontic treatment such as friction which leads to reduced force deliver therefore higher forces are being applied in-order to overcome it but it leads to undesirable effects on the teeth as well as anchorage loss therefore various methods have been implemented such as modified brackets using various coating, reduced forces, and by altering the size, shape and surface of the wire materials which helps in reduced friction during sliding mechanics. Studies have stated that tungsten disulphide (WS2) nanoparticle coated orthodontic wires and on the surface of the brackets resulted in reduced friction [30]. Studies have stated that carbone nitride, tungsten disulfide, molybdenum disulfide, diamond like carbon, nickel-phosphorus are used to coat stainless steel wire as they exert corrosion resistance, reduced friction, increased elasticity, antimicrobial property as a result there is no plaque adherence on the wire or around the brackets which prevents the formation of white spot lesions further preventing caries formation [31,32,33, 34,35].

## Antimicrobial Nanoparticles:

Orthodontic treatment is more prone to plaque accumulation due to which they lead to decay or white spot lesions as the nanoparticles has antimicrobial property, they are used to coat brackets or incorporated in composites or bonding agent some of the nanoparticles such as copper, silica, silver, ZnO, gold and titanium dioxide are being used thereby reducing the process of demineralization and improving the quality of treatment.

## Making of hollow wires:

NiTi and stainless steel are the most commonly used wires in orthodontics in which NiTi wire is used due to its property of super elasticity and shape memory they are widely used as they are hollow wires. Electro spinning method is used to coat polymer fibre with NiTi nanoparticle once it's coated the fiber is removed so that the wire is hollow one of the major advantages is lesser material is required for manufacturing the wire and the another is that as the wire is hollow it exerts super elasticity and shape memory [36].

## Chitosan nanoparticles:

Chitosan nanoparticles have antibacterial property therefore they are used in resin base composites therefore studies have been done in various concentration such as 1%, 5% and 10% in combination with ZnO as it had better antibacterial effect when used in combination. Studies have proved that chitosan NP when mixed with ZnO-NPs in the concentration of 10% had better antibacterial property when compared to other concentration [37].

## Fluoroapatite, fluorohydroxyapatite or hydroxyapatite NPs:

Fluorohydroxyapatite crystals have the advantage of fluoride release thereby preventing white spot lesions therefore they are been infused in GIC and used as banding cements as their particle size is smaller, they penetrate easily thereby preventing micro leakage, white spot lesion formation but reduced shear bond strength was there. Studies have proved the fluoride release increased after 70 days of its cementation [38,39].

## Silver nanoparticles:

'Silver nanoparticles are known for its antibacterial property therefore various studies have been done by incorporating in composites, orthodontic wires, brackets and bands, as they had antibacterial property, they reduced the bacterial colonisation thereby reducing the plaque formation which in turn reduces the risk of white spot lesions post orthodontic treatment. Studies have stated that when silver nanoparticles containing silica nanofillers are added to composites they showed better antibacterial property therefore further studies have been done by varying the concentration of the nanoparticle 1%,5% and 10% being added to the composite reduced the adhesion of the bacterial colonies to the wire thereby reducing the caries formation and had increased shear bond strength. It was found that 1% and 5% concentration was safer whereas 10% caused major cellular changes [40, 41]. Various studies were done using silver nanoparticle by incorporating it in brackets, resins, composites etc as they have belter antibacterial property some of the studies are such as silver nanoparticle were coated on orthodontic brackets and placed on rats saliva, plaque, blood samples and smear layer were collected and studies showed that they had antibacterial effect thereby reducing the formation of white spot lesion, silver nanoparticle was incorporated in composites it showed that it increased its storage property without any changes in shear bond strength, when coated on implants studies proved that it reduced the bacterial colonisation around the implant thereby preventing implant failure but further studies are required to study their adverse effects on tissue [42,43,44].

## Silicon Dioxide, Titanium dioxide or Silver Nanoparticles to acrylic resins:

Nanoparticles are being incorporated in acrylic resin due to its antibacterial property and less cytotoxicity some of them are silver , silicon dioxide and titanium dioxide nanoparticles which have been used in various studies but the major drawback of it was that they did not assess the toxicity of the nanoparticle in various concentration and its antibacterial efficiency but the biocompatibility was done for a shorter span of time as the particle size is smaller they can interact easily and can cause immunological changes[45,46,47,48].
